# Cell division cycle associated 5 promotes colorectal cancer progression by activating the ERK signaling pathway

**DOI:** 10.1038/s41389-019-0123-5

**Published:** 2019-02-26

**Authors:** Aling Shen, Liya Liu, Hongwei Chen, Fei Qi, Yue Huang, Jiumao Lin, Thomas Joseph Sferra, Senthilkumar Sankararaman, Lihui Wei, Jianfeng Chu, Youqin Chen, Jun Peng

**Affiliations:** 1 Academy of Integrative Medicine, Fujian University of Traditional Chinese Medicine, 1 Qiuyang Road, Minhou Shangjie, Fuzhou, Fujian 350122 China; 2 Fujian Key Laboratory of Integrative Medicine on Geriatrics, Fujian University of Traditional Chinese Medicine, 1 Qiuyang Road, Minhou Shangjie, Fuzhou, Fujian 350122 China; 3 Department of Pediatrics, Case Western Reserve University School of Medicine, Rainbow Babies and Children’s Hospital, Cleveland, OH 44106 USA

## Abstract

Cell division cycle associated 5 (CDCA5) is implicated in the development and progression of a variety of human cancers. Functional significance of CDCA5 in colorectal cancer (CRC), however, has not been investigated. Using a combination of on-line data mining, biochemistry, and molecular biology, we examined the potential oncogenic activity of CDCA5 and the underlying mechanisms. Experiments with human tissue sample showed increased CDCA5 expression in CRC vs. in noncancerous adjacent tissue, and association of CDCA5 upregulation in CRC tissues with shorter patient survival. Also, representative CRC cell-lines had higher CDCA5 expression vs. fetal colonic mucosal cells. CDCA5 knockdown using lentivirus-mediated shRNA inhibited the proliferation and induced apoptosis in cultured HCT116 and HT-29 cells, and suppressed the growth of xenograft in nude mice. CDCA5 knockdown decreased the expression of CDK1 and CyclinB1, increased caspase-3 activity, cleaved PARP and the Bax/Bcl-2 ratio. CDCA5 knockdown also significantly decreased phosphorylation of ERK1/2 and expression of c-jun. Taken together, these findings suggest a significant role in CRC progression of CRC, likely by activating the ERK signaling pathway.

## Introduction

Colorectal cancer (CRC) is the third leading cause of cancer-related death worldwide^1^. Despite recent advances in early diagnosis of and treatments for CRC, patient mortality remains high.

Uncontrolled growth is a key feature of cancers^2,3^. Accordingly, suppressing the proliferation of cancer cells represent an important strategy in anticancer treatment. In eukaryotic cells, proliferation is primarily regulated by cell cycle^4^ that contains three major checkpoints—one at the G1–S transition and two at G2–M transition^5^. Sister chromatid cohesion in the S phase and segregation of sister chromatids in the anaphase of mitosis are two important processes during cell mitosis that safeguard the accurate separation of parental chromosomes into two daughter cells. Human CDCA5 (cell division cycle associated 5), also known as sororin, was originally identified as a substrate of the anaphase-promoting complex^6–8^. CDCA5 is required for stable binding of cohesin to chromatid in the S and G2/M phases and is degraded through anaphase-promoting complex-dependent ubiquitination in the G0/G1 phase^6–9^.

CDCA5 has been found to be overexpressed, and correlated with poor prognosis in several human cancers, including lung carcinomas, urothelial carcinoma, and oral squamous cell carcinoma^10–14^. Consistent with CDCA5 overexpression in cancer cells, knockdown of CDCA5 could inhibit cancer growth by arresting the cell cycle in the G2/M phase and promoting apoptosis^11,14^.

In the current study, we examined whether CDCA5 is also implicated in the development and progression of CRC. First, we compared gene-expression profile in primary CRC lesions vs. matched healthy tissues. Analysis of the differentially expressed genes using RNA interference and high-content screening identified CDCA5 as a potential target. We then conducted a series of experiments using representative CRC cell lines as well as xenograft nude mice models to examine the functional role of CDCA5.

## Results

### CDCA5 is highly expressed in CRC tissues and cultured cells

**Fig. 1:**
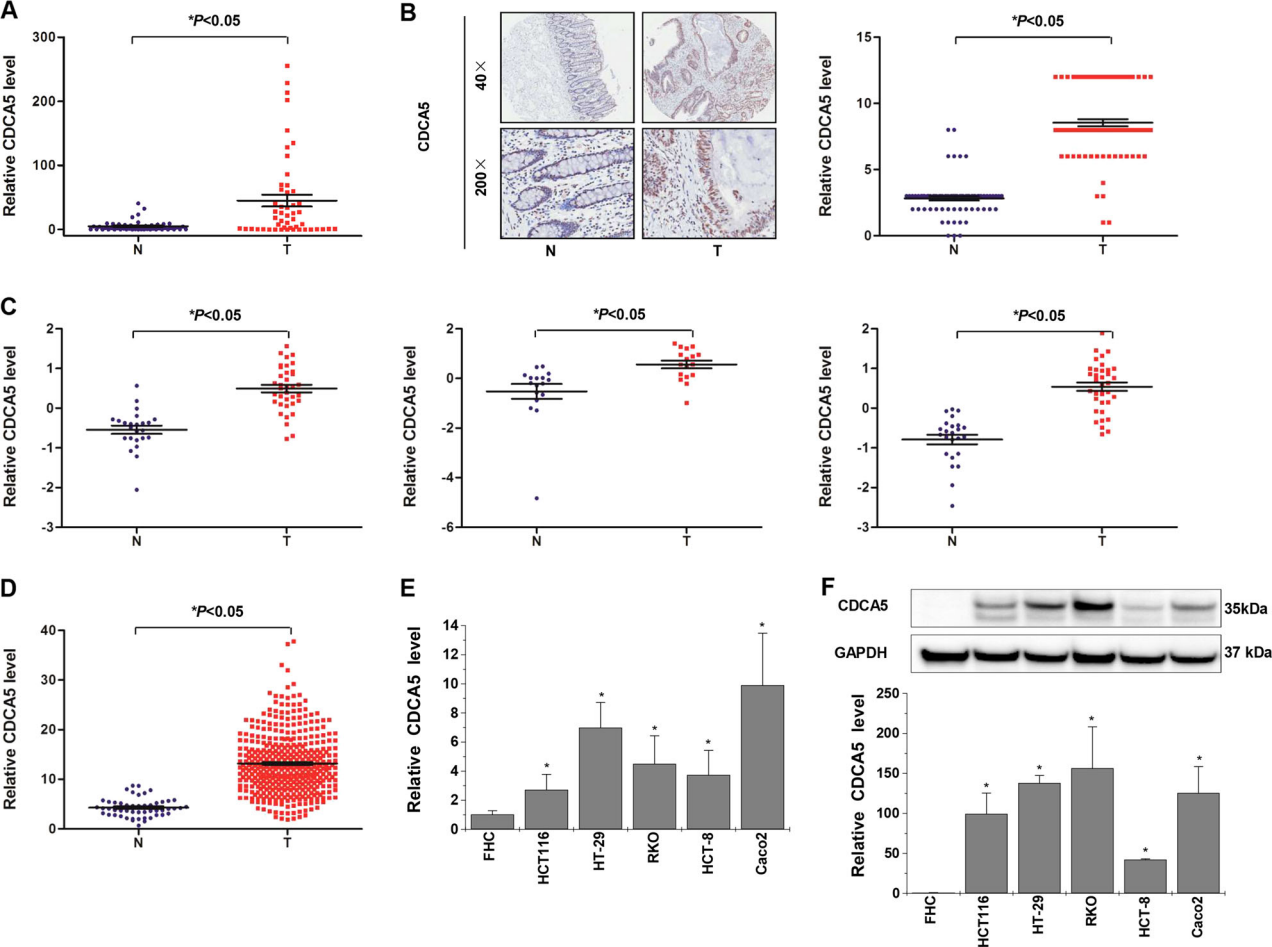
CDCA5 is overexpressed in human CRC tissues and cultured CRC cells. **a** CDCA5 mRNA in tissues from 50 CRC patients, as analyzed by q-PCR. GAPDH was used as internal control. **b** CDCA5 protein in 73 pairs of CRC tissues and adjacent normal tissues, as determined by IHC-based tissue microarray. Image magnification at ×40 or ×200. T: tumor tissues; N: normal tissues. ^*^P < 0.05, tumors vs. normal tissues. **c**, **d** CDCA5 mRNA in the R2 Bioinformatic Platform (**c**) and TCGA (**d**). ^*^P < 0.05, tumors vs. normal tissues. **e** CDCA5 mRNA and **f** CDCA5 protein in a panel of CRC cell lines and FHC cells, as determined by q-PCR and Western-blot analyses. GAPDH was used as the internal control. *n* = 3. ^*^P < 0.05, vs. FHC cells. Data are shown as mean ± SD

Quantitative real-time polymerase chain reaction (qPCR) assay in 50 pairs of primary CRC lesions and adjacent noncancerous tissues revealed higher CDCA5 mRNA level in CRC tissue (Fig. 1a). Such result was verified by immunohistochemical (IHC)-based tissue microarray (TMA) of 73 pairs of primary CRC lesions and adjacent noncancerous tissue (Fig. 1b). Similar results were obtained with online data mining using the R2 Bioinformatic Platform (http://r2.amc.nl) and TCGA (https://cancergenome.nih.gov/) (Fig. 1c, d). qPCR and Western-blot analyses of cultured human CRC cell lines (Caco-2, HT-29, RKO, HCT116, and HCT-8) also showed significantly higher CDCA5 expression in CRC cells than in fetal colonic mucosal cells (FHC) (Fig. 1e, f; *P* < 0.05).

### CDCA5 overexpression is associated with poor prognosis in CRC patients

**Fig. 2:**
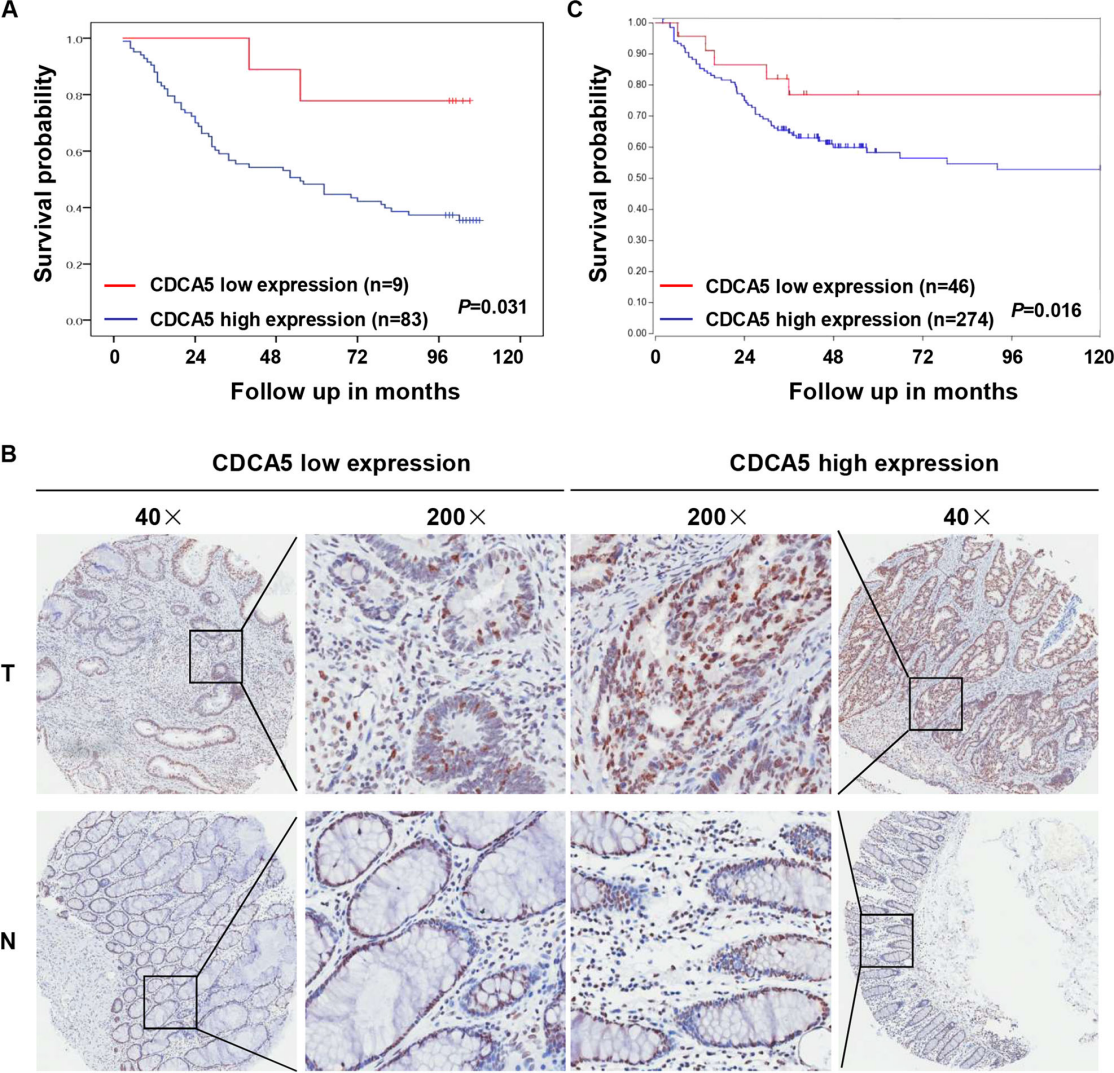
Association between CDCA5 overexpression and poor patient prognosis. **a** Correlation between CDCA5 protein (IHC-based TMA) and patient survival, was analyzed with Kaplan–Meier plots in 92 CRC patients (*P* < 0.05). **b** Representative images of high- or low-CDCA5 expression. Magnification at ×40 or ×200. **c** Survival analysis based on CDCA5 expression in 320 CRC patients (GEO ID: GSE24551) from a public clinical microarray dataset R2 bioinformatic platform (*P* < 0.05). Survival was analyzed with log-rank test

Analysis of 92 CRC patients showed higher overall survival rate in subjects with higher CDCA5 expression (as determined with IHC-based TMA) (Fig. 2a, b; *P* < 0.05). High-CDCA5 expression was also associated with more advanced N stage (Supplementary Table 1; *P* < 0.05). Online data mining using the R2 Bioinformatic Platform (http://r2.amc.nl) also revealed an association between high-CDCA5 expression and poor overall survival in CRC patients (Fig. 2c; *P* < 0.05).

### CDCA5 knockdown inhibits the proliferation of cultured CRC cells

We next constructed three lentiviral shRNAs specific for CDCA5. qPCR and Western-blot analyses revealed significantly reduced CDCA5 mRNA (Supplementary Fig. 1A, B) and protein (Supplementary Fig. 1C) levels with all three shRNAs in both HCT116 cells and HT-29 cells (constitutively expressing CDCA5; Fig. 1e, f). The sh-CDCA5-2 construct was used for subsequent experiments, and referred to as “sh-CDCA5”.

**Fig. 3:**
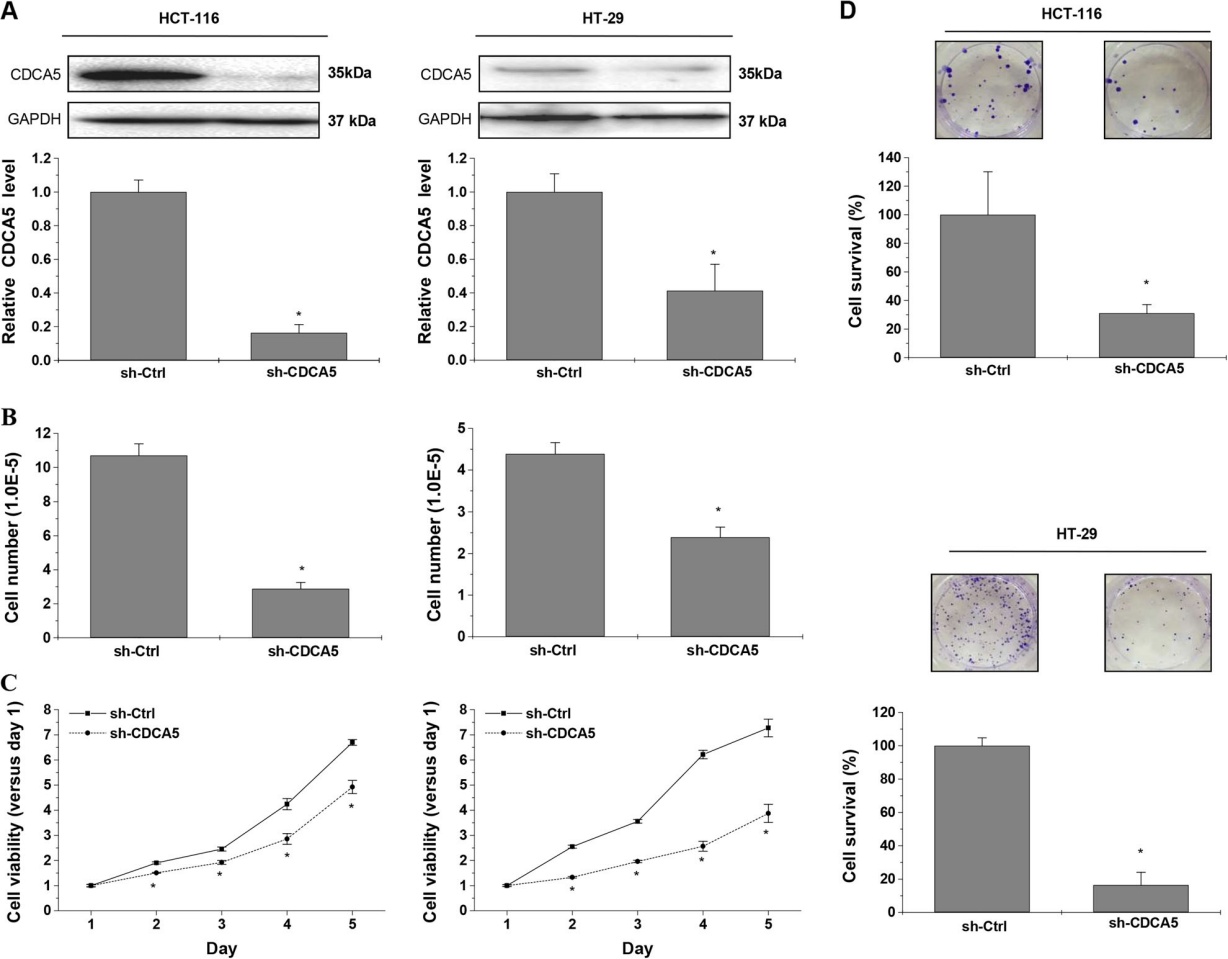
CDCA5 knockdown suppresses the growth of cultured CRC cells. **a** Western-blot analysis of CDCA5 in HCT116 (left panel) and HT-29 (right panel) cells after transduction with sh-CDCA5 vs. sh-Ctrl lentivirus. The integrated band density was determined using the ImageLab Software, and GAPDH as the internal control (^*^P < 0.05). **b** After transduction with sh-CDCA5 vs. sh-Ctrl lentivirus for 72 h, cell number was counted using trypan blue exclusion (^*^P < 0.05, vs. sh-Ctrl). Left: HCT116; Right: HT-29. **c** Cell viability of HCT116 and HT-29 cells was determined using CCK-8 assay after transduction with sh-CDCA5 vs. sh-Ctrl lentivirus. Data were normalized to viability on day 1 and shown as fold change. ^*^P < 0.05 vs. sh-Ctrl. **d** Cell survival was analyzed by colony formation assay in HCT116 or HT-29 cells after transduction with sh-CDCA5 or sh-Ctrl lentivirus. Representative images of colonies after CDCA5 knockdown are shown. ^*^P < 0.05 vs. sh-Ctrl. All experiments were performed in triplicate

**Fig. 4:**
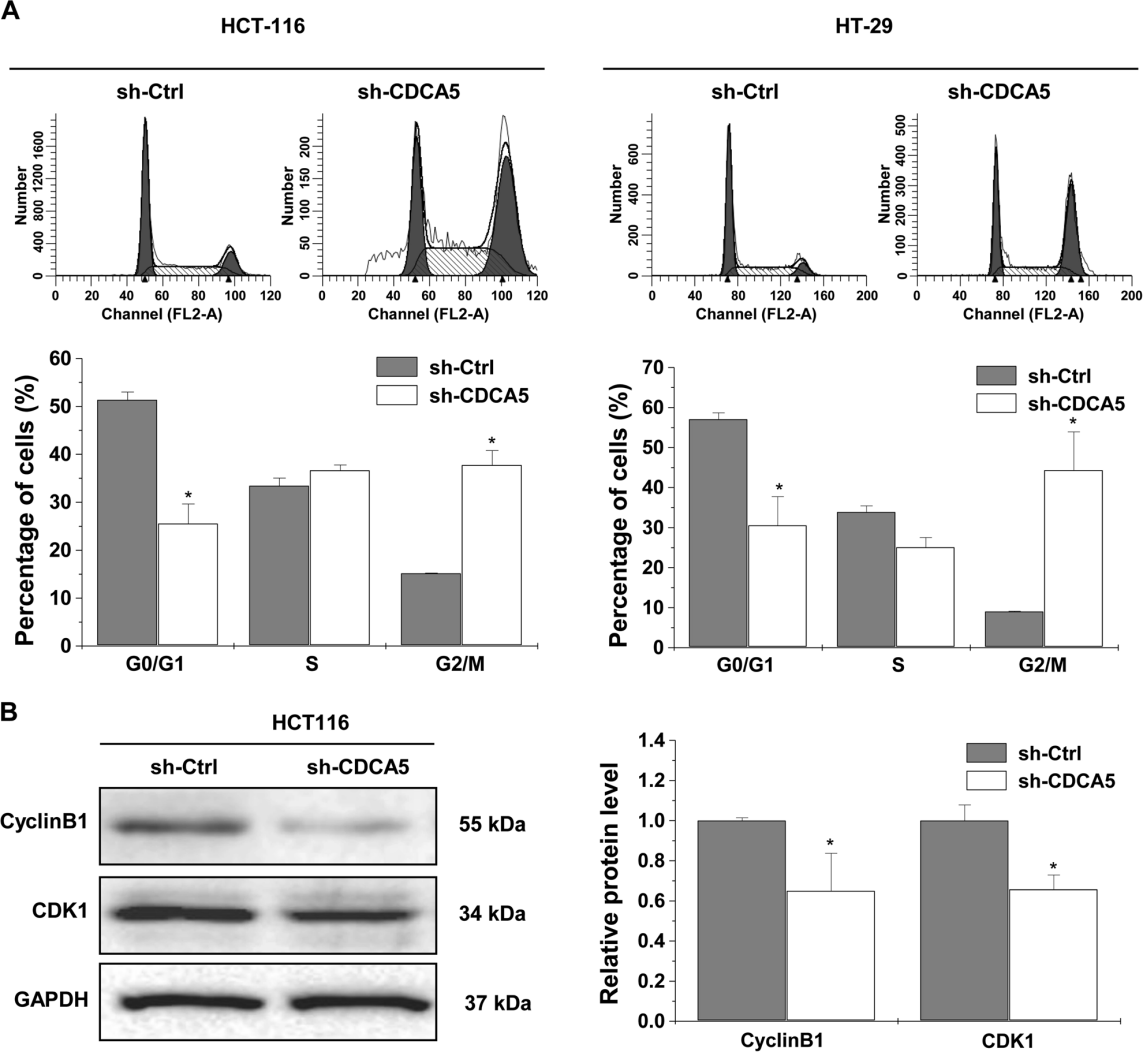
CDCA5 knockdown induces cell cycle arrest. **a** Cell cycle distribution was determined by flow cytometric analysis in HCT116 (left panel) and HT-29 cells (right panel) after CDCA5 knockdown. The percentage of cells in the G0/G1, S, and G2/M phases was calculated. ^*^P < 0.05 vs. sh-Ctrl. **b** Western-blot analysis of CDK1 and CyclinB1 in HCT116 cells after transduction with sh-CDCA5 or sh-Ctrl lentivirus. The integrated band density was determined by ImageLab Software, and GAPDH as the internal control (^*^P < 0.05). All experiments were performed in triplicate

CDCA5 knockdown in cultured HCT116 and HT-29 cells significantly reduced cell number (Fig. 3b; *P* < 0.05 vs. sh-Ctrl), cell viability (Fig. 3c; *P* < 0.05) and colony formation (Fig. 3d; *P* < 0.05). Cell cycle analysis showed decreased cell percentage in the G0/G1 phase and increased percentage in the G2/M phase in both HCT116 and HT-29 cells (Fig. 4a; *P* < 0.05 vs. sh-Ctrl). The expression of G2/M-related proteins CDK1 and CyclinB1 was decreased by CDCA5 knockdown in HCT116 cells (Fig. 4b).

### CDCA5 knockdown induces apoptosis in cultured CRC cells

**Fig. 5:**
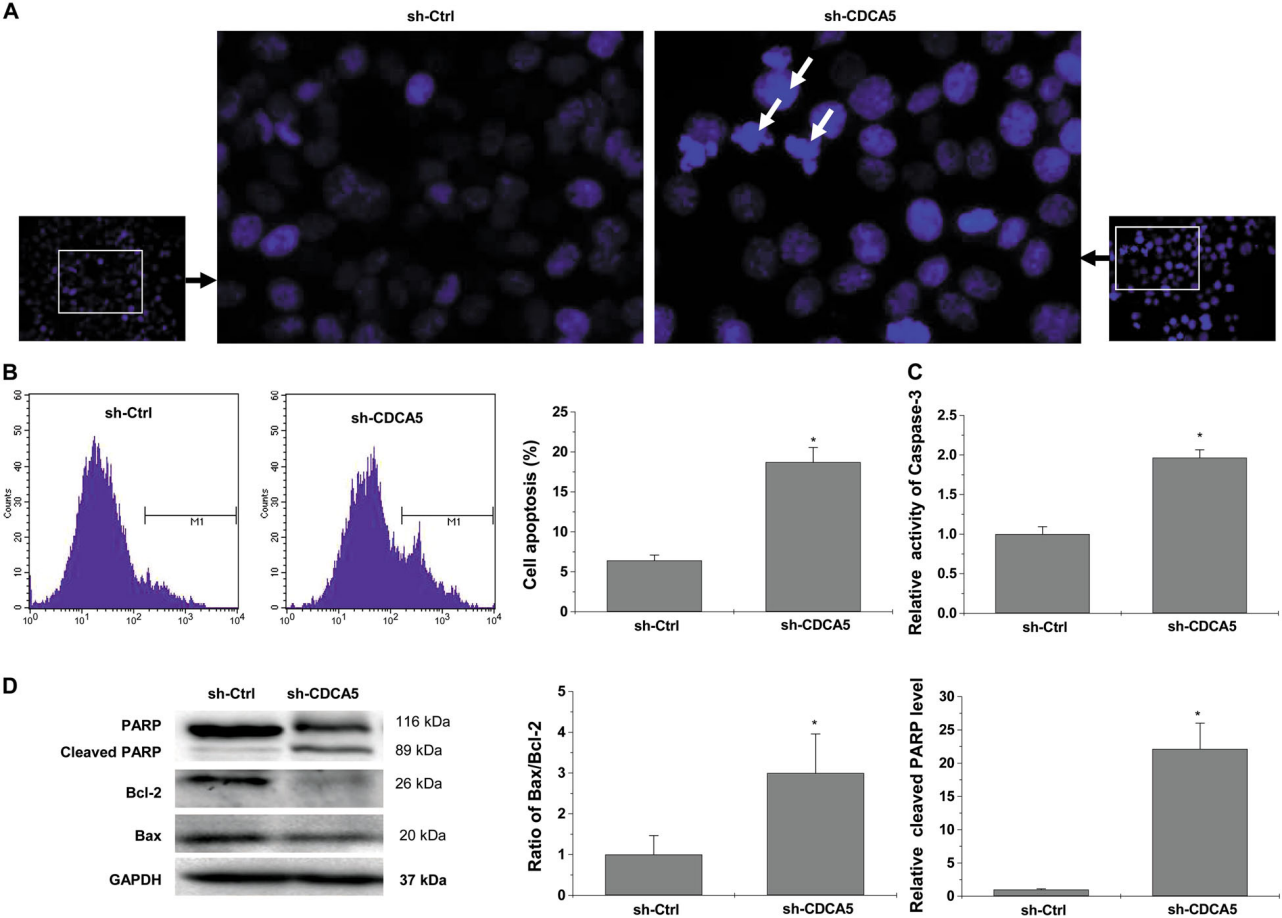
CDCA5 knockdown induced cell apoptosis in colorectal cancer cells in vitro. **a** Hoechst staining was performed to determine the cell apoptosis of HCT116 cells after CDCA5 knockdown (×400 magnification). White arrows indicate cells with morphologic changes characteristic of apoptosis. **b** Annexin V-APC staining followed by flow cytometric analysis in HCT116 cells after CDCA5 knockdown. ^*^P < 0.05, vs. sh-Ctrl. **c** Caspase-3 activity was determined using a colorimetric assay in HCT116 cells after CDCA5 knockdown, ^*^P < 0.05, vs. sh-Ctrl. **d** Protein levels of BAX, Bcl-2, and PARP in HCT116 cells after transduction with sh-CDCA5 vs. sh-Ctrl lentivirus, as determined by Western-blot analysis. The integrated band density was determined using ImageLab Software, and GAPDH as the internal control. ^*^P < 0.05 vs. sh-Ctrl. All experiments were performed in triplicate

Hoechst staining revealed typical apoptotic morphological features (e.g., condensed chromatin and fragmented nuclei) in HCT116 cells upon CDCA5 knockdown (Fig. 5a). Flow cytometry with Annexin V staining increased the percentage of apoptotic cells (Fig. 5b; *P* < 0.05 vs. sh-Ctrl). Caspase-3 activity was increased (Fig. 5c; *P* < 0.05 vs. sh-Ctrl). Western-blot analysis showed increased Bax/Bcl-2 ratio and increased expression of cleaved Poly ADP-ribose polymerase (PARP) (Fig. 5d; *P* < 0.05 vs. sh-Ctrl).

### CDCA5 suppresses the growth of CRC tumor xenograft

**Fig. 6:**
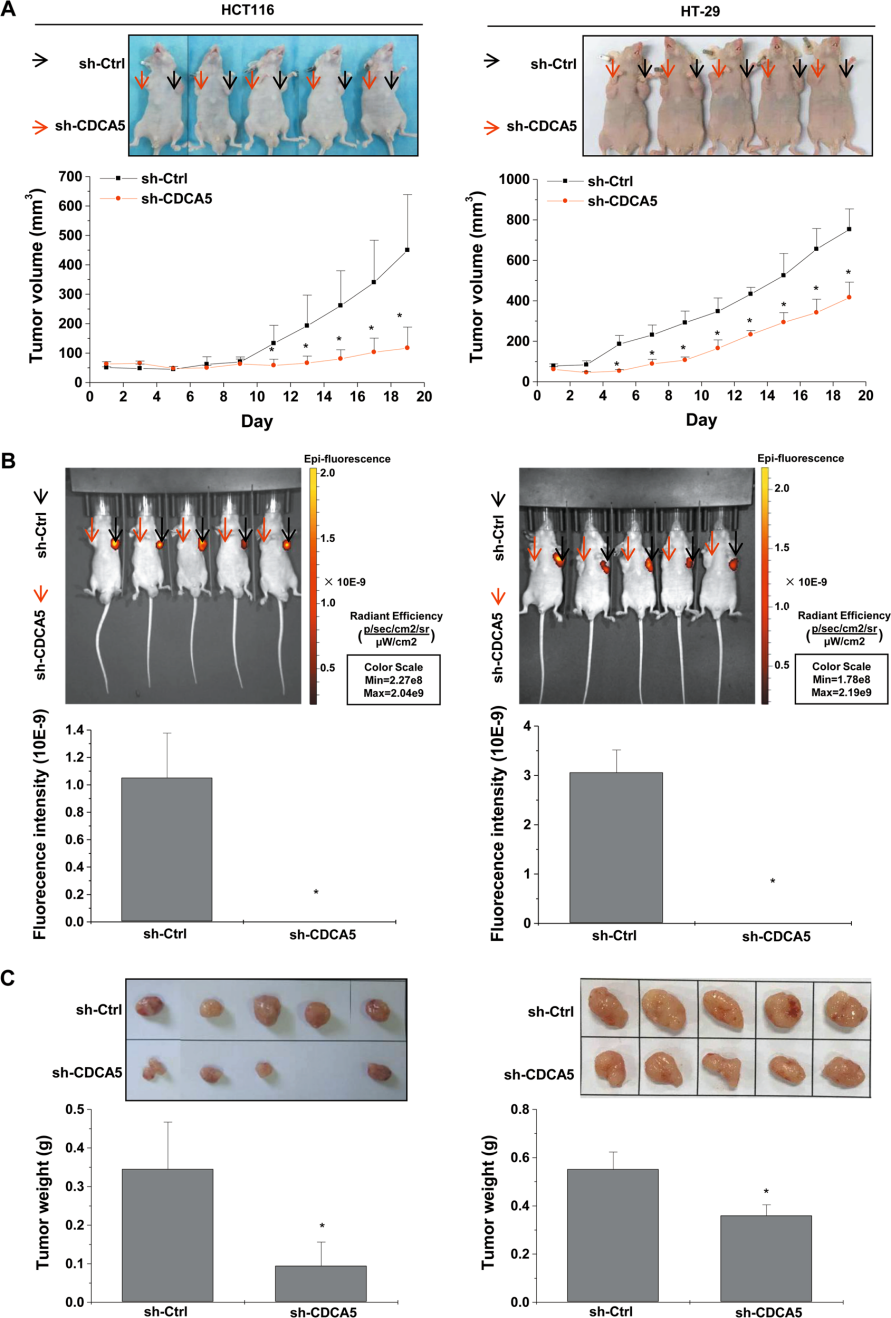
CDCA5 knockdown suppresses tumor growth in vivo. A xenograft nude mouse model was used to investigate the effect of CDCA5 knockdown on tumor growth. HCT116 or HT-29 cells were transduced with sh-CDCA5 or sh-Ctrl lentivirus, and then injected subcutaneously into BALB/c nude mice. **a** Average tumor volume from day 5 after injection (lower panel; ^*^P < 0.05 vs. sh-Ctrl). **b** Representative images of fluorescence (upper panel) and GFP intensity (lower panel; ^*^P < 0.05 vs. sh-Ctrl) were observed at the end of experiments. **c** Representative images of tumor (upper panel) was observed and average tumor weight (lower panel) were assessed (^*^P < 0.05 vs. sh-Ctrl group)

**Fig. 7:**
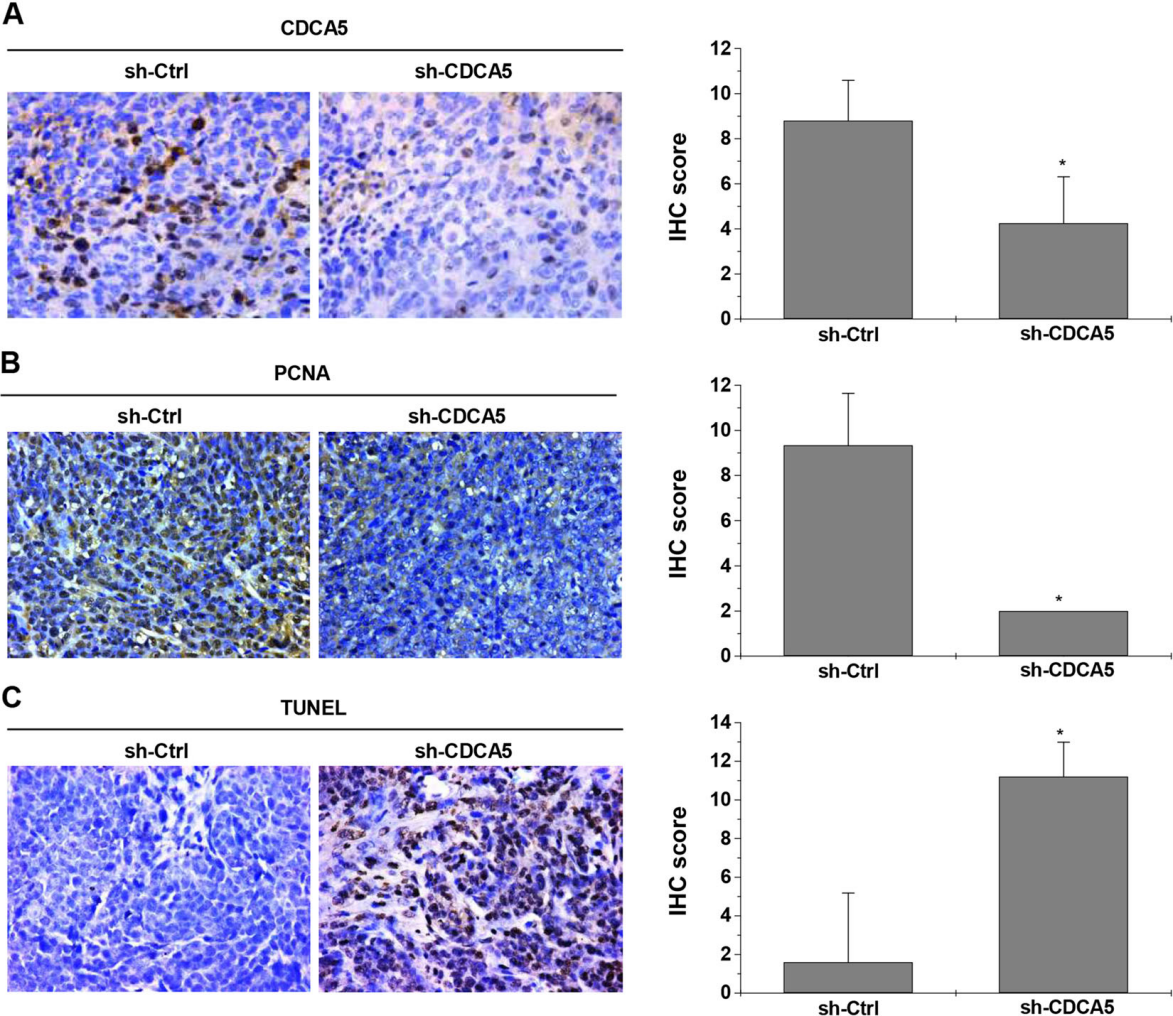
CDCA5 knockdown inhibits cell proliferation and induces cell apoptosis in vivo. IHC was performed to detect CACA5 (**a**) and PCNA (**b**) expression in tumor sections, and TUNEL assay (**c**) was used to determine the apoptotic cells in tissues. The representative images of IHC or TUNELA assay (left panel, ×400) and statistical analysis of CDCA5 protein expression (right panel) are shown (^*^P < 0.05 vs. sh-Ctrl group). All experiments were performed in triplicate

Upon subcutaneous implantation, HCT116 xenograft transduced with sh-CDCA5 grew at a much lower rate vs. the control (Fig. 6a for tumor volume; Fig. 6b for intratumoral green fluorescent protein (GFP) fluorescence and Fig. 6c for tumor weight; *P* < 0.05 for all). IHC showed that CDCA5 knockdown (Fig. 7a; *P* < 0.05 vs. sh-Ctrl) significantly decreased proliferating cell nuclear antigen (PCNA) expression in xenograft tumor (Fig. 7b; *P* < 0.05 vs. sh-Ctrl). Terminal deoxynucleotidyl transferase dUTP nick end labeling (TUNEL) assay showed increased cell apoptosis in HCT116 xenograft upon CDCA5 knockdown (Fig. 7c; *P* < 0.05 vs. sh-Ctrl).

### CDCA5 knockdown suppresses the activation of ERK signaling pathway

**Fig. 8:**
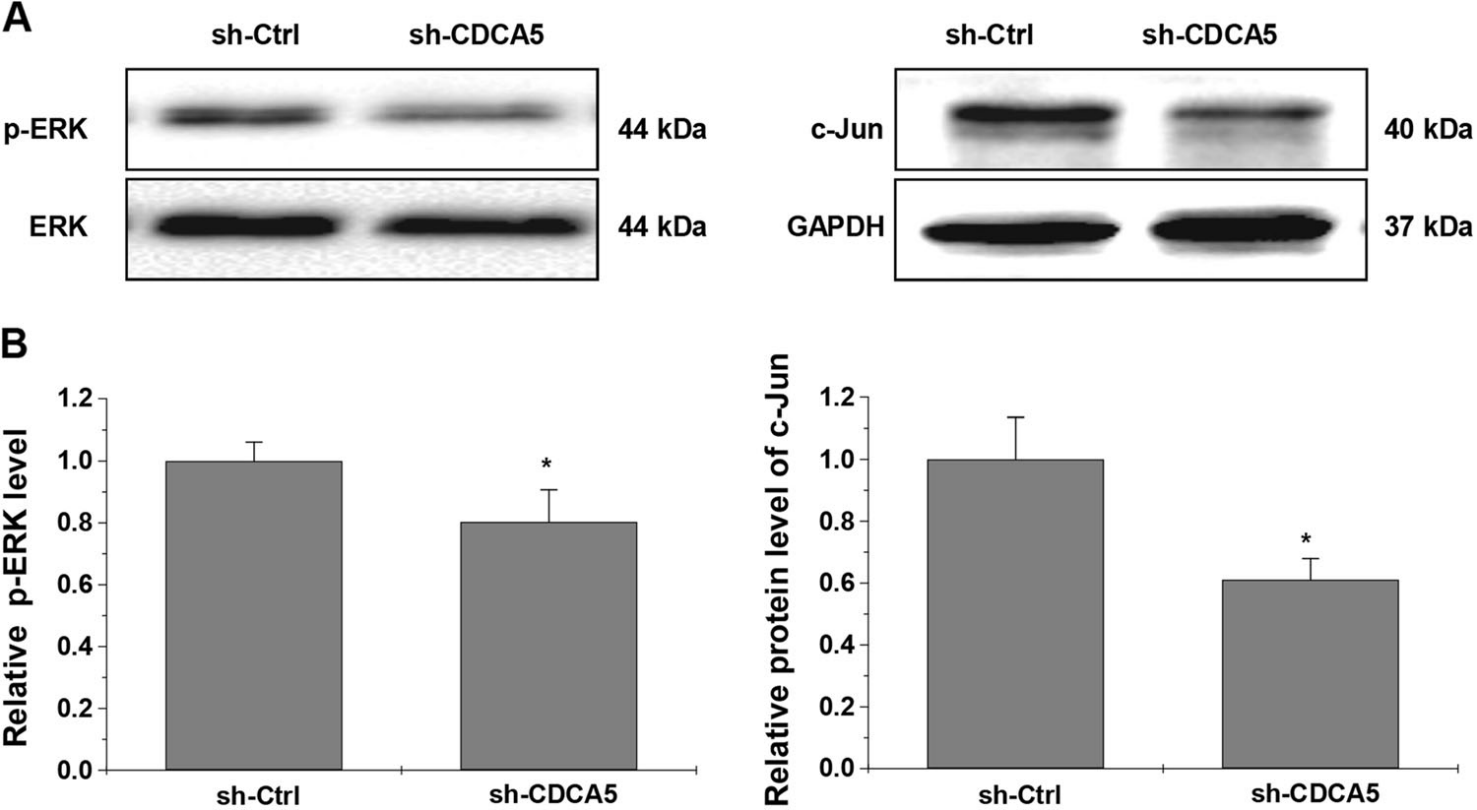
CDCA5 knockdown suppresses activation of ERK signaling pathway. **a** Protein levels of p-ERK, ERK, and c-jun in HCT116 cells after transduction with shRNA-CDCA5 or sh-Ctrl lentiviurs, as determined by Western-blot analysis. **b** The integrated band density was determined using ImageLab Software, using GAPDH as the internal control. Gene expression is presented as the percentage relative to the sh-Ctrl group (^*^P < 0.05). All experiments were performed in triplicate

Activation of the ERK pathway plays a pivotal role in the pathogenesis of CRC^15–18^. In our experiment, CDCA5 knockdown significantly decreased the phosphorylation of ERK1/2 and c-jun expression in cultured HCT116 cells (Fig. 8; *P* < 0.05).

## Discussion

The first major finding in the current study is the overexpression of CDCA5 in CRC tissues vs. in adjacent noncancerous tissues. The overexpression is apparent in our own study cohort and supported by the online datasets in the R2 application website and TCGA. Overexpression of CDCA5 was also found in several representative CRC cell lines vs. in normal human FHC, suggesting that CDCA5 overexpression is a common event in CRC. Survival analysis based on TMA of CRC samples and online public database associated higher CDCA5 expression with poorer patient prognosis. These findings are largely consistent with the studies in lung carcinoma, urinary bladder carcinoma, and oral squamous cell carcinoma^11,13,14^, and suggested a functional role for CDCA5 for a broader range of cancers. The results must be interpreted with caution due to the relatively small sample size and the inclusion of only Chinese patients. The prognostic significance of CDCA5 requires further validation by studies of larger sample size with diverse ethnic background.

Previous studies indicated that CDCA5 knockdown could inhibit the growth of lung carcinoma and oral squamous cell carcinoma cell^11,14^. In the current study, we knocked down the expression of CDCA5 in two representative CRC cell lines (HCT116 and HT-29) using a lentivirus-mediated shRNA, and found decreased cell growth in vitro as evidenced by decreased cell number, viability, and survival rate. Using a nude mouse xenograft model bearing HCT116 or HT-29 cells transduced with sh-CDCA5 lentivirus, we found that CDCA5 knockdown could markedly decrease tumor growth. Taken together, these findings suggest an oncogenic activity of endogenous CDCA5 in CRC.

Proliferation of eukaryotic cell is primarily mediated by cell cycle. G2/M progression, one of the main checkpoints, is closely modulated by the protein complex of CyclinB1/CDK1^19,20^. CDCA5 is required for stable binding of cohesion to chromatid in the G2/M phase and plays an essential role in cell cycle regulation^6–9^. Consistent with a previous study in oral squamous cell carcinoma^14^, we found blocked G2/M progression upon CDCA5 knockdown in both HCT116 and HT-29 cells. CDCA5 knockdown also significantly decreased CyclinB1 and CDK1 expression. Mitochondrion-dependent apoptosis is a major apoptotic pathway. PARP, a protein essential for DNA repair, could be cleaved by caspases during the process of apoptosis. Accordingly, cleaved PARP is commonly used as an indicator of apoptosis. Bcl-2 family members, including antiapoptotic Bcl-2 and proapoptotic Bax^21,22^, are critical mediators of mitochondrion-dependent apoptosis. Aberrant expression of Bcl-2 family proteins is commonly found in cancers. In this study, we found that CDCA5 knockdown-induced apoptosis in HCT116 cells. Colorimetric assay and western-blot analysis showed increased caspase-3 activity, PARP expression and Bax/Bcl-2 ratio in HCT116 cells in response to CDCA5 knockdown. IHC analysis and TUNEL assay in xenograft models confirmed the suppression of cell proliferation and promotion of cell apoptosis by CDCA5 knockdown. These findings indicate that CDCA5 produces oncogenic activity by disrupting the balance of proliferation/apoptosis in cancer cells. However, underlying mechanism of CDCA5 knockdown on suppression tumor growth of CRC cells need to be explored in future studies.

The ERK signaling pathway plays an important role in a variety of cellular processes, including cell survival, cell differentiation, apoptosis, invasion, and inflammation^18,19,23,24^. Activation of the ERK pathway is one of the key mechanisms for the initiation and progression in many human cancers^15–18^, including CRC^25–28^. Our finding that CDCA5 knockdown decreased the phosphorylation level of ERK1/2 and expression of c-jun in CRC cells suggests that activation of the ERK pathway contributes to the oncogenic activities of CDCA5 in CRC. However, potential effects of CDCA5 on activation of p38MAPK, JNK, and other pathways need to be further addressed.

In summary, we found CDCA5 overexpression in CRC and an association of CDCA5 overexpression with poor patient survival. Silencing CDCA5 expression suppressed the tumor growth in vitro and in vivo, possibly by inhibiting the ERK signaling. These studies highlighted the biological function of CDCA5, and suggested that CDCA5 could be used as a potential biomarker in CRC.

## Methods

### Patients and specimens

A total of 50 pairs of primary CRC lesions and matched noncancerous tissues (5 cm away from the margin of cancerous tissues) were collected from CRC patients, who received resection at First Hospital Affiliated to Fujian University of Traditional Chinese Medicine (FJTCM) or Fujian Provincial Hospital in 2013 or 2014. None of the patients received chemotherapy or radiotherapy prior to the surgery. Tissue specimens were either fixed in 4% paraformaldehyde for 24 h followed by paraffin embedding, or snap frozen and kept in liquid nitrogen until further use. Clinicopathologic characteristics of the patients are shown in Supplementary Table 2. The study was approved by the Ethics Committee of FJTCM and Fujian Provincial Hospital. Written informed consent was obtained from all human participants.

### q-PCR analysis

Total RNA was extracted using RNAiso Plus reagent (Takara; Dalian, Liaoning, China), and reverse-transcribed (1 μg) into cDNA using PrimeScript RT kit (Takara). Gene expression was examined using q-PCR with ABI 7500 Fast Real-Time PCR System (Applied Biosystems; Carlsbad, CA, USA), as described previously^29^. Sequence of the primers are shown in Supplementary Table 3. mRNA was quantified using 2^−^^ΔΔCT^ ^29^.

### TMA and survival analysis

TMA chips of CRC and control tissues were obtained from Shanghai Outdo Biotech Company (Shanghai, China; Cat#: HColA180Su09). CDCA5 IHC was conducted using an antibody against CDCA5 (Rabbit monoclonal to CDCA5-C-terminal; dilution 1:800; Abcam, ab192237; USA), as described previously^30^. Scoring was carried out by two experienced pathologists blinded to tissue identity using a grading system based on staining intensity (no staining, 0; weak, 1; moderate, 2; strong, 3) and percentage of positive-staining cells (1–25% positive, 1; 26–50%, 2; 51–75%, 3; 76–100%, 4)^31^. The final score was calculated as intensity score × percentage score. For survival analysis, CDCA5 expression in CRC tissues was classified into low (final score: 0–6) vs. high (final score: 7–12). Kaplan–Meier survival curves were plotted for high- and low-expression groups and analyzed using log-rank test.

### Bioinformatics analysis

CDCA5 mRNA in CRC vs. control tissues was analyzed using the R2 Bioinformatic Platform (http://r2.amc.nl)^32–34^ and TCGA (https://cancergenome.nih.gov/). Kaplan–Meier analysis was performed to analyze the correlation between CDCA5 mRNA and patient survival in a dataset of 320 subjects (GEO ID: GSE24551) through R2 web application (http://r2.amc.nl)^35^.

### Cell lines and cell culture

CRC cell lines (RKO, HCT-8, HT-29, HCT116, and Caco-2) were purchased from Cell Bank, Shanghai Institutes for Biological Sciences, Chinese Academy of Sciences (Shanghai, China). FHC (normal human colon cell line) was purchased from American Type Culture Collection (Manassas, VA, USA). RKO, HCT116, and HCT-8 were grown in RPMI-1640 (Gibco; Carlsbad, CA, USA), HT-29 in M5’A (KeyGEN; Jiangsu, China), Caco-2 in DMEM (Gibco), and FHC in DMEM:F12 (Gibco), at 37 ℃ in a humidified atmosphere of 5% CO_2_. All culture media contained 10% fetal bovine serum (FBS) (Gibco), 100 units/ml penicillin and 100 mg/ml streptomycin (Hyclone; Logan, UT, USA).

### Lentivirus transduction

Three independent shRNA targeting CDCA5 (sh-CDCA5-1, 2, 3-lentivirus-GFP; Cat#: PIEL248072721) and a sh-Ctrl (sh-Ctrl-lentivirus-GFP, GV248; Cat#: LVCON077) were provided by Shanghai GeneChem Company (Shanghai, China). After cells were seeded in six-well plates, lentivirus encoding shRNA or control shRNA was added at the multiplicity of infection recommended by the manufacturer (MOI: 10). After 72-h incubation, CDCA5 expression was determined using q-PCR and Western blot.

### Trypan blue exclusion and cell number counting

Cells were stained using 0.4% trypan blue, and analyzed using a Countstar Automated Cell Counter (Inno-Alliance Biotech, Inc.; Wilmington, DE, USA).

### CCK-8 assay

Cell viability was examined using the Cell Counting Kit-8 (Dojindo; Japan). Incubation lasted for 2 h at 37 ℃. Absorbance was detected at 450 nm using Infinite 200 Pro microplate reader (Tecan; Männedorf, Switzerland).

### Colony formation assay

Cells were seeded into 12-well plates (500 cells/well) and incubated in humidified air containing 5% CO_2_ at 37 °C for 10–12 days. Culture medium was replaced every 2–3 days. The formed colonies were washed with phosphate-buffered saline, fixed with 4% formaldehyde and then stained with 0.01% crystal violet. The number of colonies was counted manually.

### Cell cycle analysis

Cells were fixed with 70% ethanol at 4 °C for 12–16 h, and stained with propidium iodide (PI; Thermo Fisher; Carlsbad, CA, USA). Percentage of cells in different cell cycle phases was analyzed using a FACS Caliber (Becton-Dickinson; San Jose, CA, USA).

### Hoechst33342 staining

Cells were fixed with 4% formaldehyde and then stained with Hoechst dye (Beyotime, Jiangsu, China) for 15–20 min. Images were captured using a phase-contrast fluorescent microscope (Leica Microsystems; Wetzlar, Germany) at ×400 magnification.

### Apoptosis analysis

Cells were incubated with Annexin V-APC solution (KeyGEN) for 15 min. The percentage of apoptotic was analyzed by flow cytometry (FACS Caliber, Becton-Dickinson).

### Caspase-3 activation analysis

Caspase-3 activity was measured using a colorimetric assay (KeyGEN). Briefly, cell lysate (100-μg protein) was mixed with 50-μl specific caspase-3 substrate (Asp-Glue-Val-Asp (DEAD)-pNA), and incubated for 2 h. Absorbance was measured at 405 nm. Caspase activity was presented relative to the control cells.

### In vivo experiments

BALB/c nude mice (male, 4–6 weeks of age,) were purchased from Shanghai SLAC Laboratory Animal Co. (Shanghai, China) and maintained in a specific pathogen-free facility. After transduction with sh-Ctrl or sh-CDCA5 lentivirus, CRC cells (1 × 10^6^) suspended in 100-µl FBS-free medium containing 50% matrigel were injected subcutaneously into the opposite flanks of mice (*n* = 5; self-control). Tumor growth was monitored every other day from day 5 after injection using a standard caliper. Tumor volume (mm^3^) was calculated as: 1/2(length × width^2^), whereas length is the longest longitudinal diameter and width is the longest transverse diameter.

At the end of experiments, mice were anesthetized with isoflurane and fluorescent images of the tumors were obtained with an IVIS spectrum whole live-animal imaging system (PerkinElmer; Santa Clara, CA, USA). The mice were sacrificed and tumor tissues were collected, weighed, and processed for IHC staining. All animal experiments were approved by the Animal Committee of FJTCM.

### Western-blot analysis

Western-blot analysis was performed as described previously^36,37^. Cell lysate was resolved by sodium dodecyl sulfate polyacrylamide gel electrophoresis (10%) and then transferred onto nitrocellulose membranes. After blocking with 5% nonfat milk for 2 h, the membranes were incubated with a primary antibody (dilution 1:1000) overnight at 4 ℃. All antibodies were from Cell Signaling Technology (CST; Beverly, MA, USA), except for CDCA5 and CDK1 (Abcam; Cambridge, MA, USA). After extensive washing, the membranes were incubated with a goat anti-rabbit HRP (horseradish peroxidase) secondary antibody (dilution 1:2000). Protein bands were detected with a chemiluminescence kit (Thermo Fisher), and analyzed using the ImageLab software. The expression of target proteins was normalized against GAPDH and presented as percentages of the control cells (sh-Ctrl or FHC cells).

### Immunohistochemistry

Immunohistochemistry was performed as described previously^38,39^. Briefly, tissue sections were incubated with an antibody against CDCA5 (1:800 dilution; Abcam, ab192237) or PCNA (1:800 dilution; Abcam, ab18197). Background was determined by omitting the primary antibody. CDCA5 expression was determined using a scoring system described in detail in the Section “Tissues microarray and survival analysis”.

### TUNEL assay

Apoptotic cells in tissue sections were detected using TUNEL staining. The percentage of TUNEL-positive cells and staining intensity were evaluated using a scoring system described in detail in the Section “Tissues microarray and survival analysis”.

### Statistics analysis

All statistical analyses were conducted using SPSS 20.0 (SPSS Inc.). Continuous variables were analyzed using Student’s *t* test for independent or paired samples as appropriate for experiments involving two groups, and with one-way ANOVA for experiments involving three or more groups, and presented as mean ± standard deviation. Survival data were analyzed using the Kaplan–Meier method and compared with log-rank test. *P* < 0.05 (two-sided) was considered statistically significant.

## Supplementary information

Supplementary metarial file.

Supplementary Figure 1.
