# Supplementary material for: Cell division cycle associated 5 promotes colorectal cancer progression by activating the ERK signaling pathway
Source: Oncogenesis. 2019 Feb 26;8(3):19. doi: 10.1038/s41389-019-0123-5 (PMC6391450; doi:10.1038/s41389-019-0123-5)
Supplement: Supplementary file 1 — Supplementary metarial file. [file 41389_2019_123_MOESM1_ESM.doc]

**Supplementary Figure Legend**

**Supplementary Figure 1: Transduction of three independent CDCA5 specific shRNA lentivirus decreases the endogenous CDCA5 expression in both mRNA and protein levels.**

HCT116 cells were transfected with one of three independent shRNA lentiviruses specific for CDCA5 or sh-Ctrl. CDCA5 mRNA (A) or protein (B) expression was determined Q-PCR or Western-blot analysis. GAPDH was used as internal control. The integrated density of protein band was assessed by ImageLab Software, the expression of CDCA5 is shown relative to sh-Ctrl (**P*<0.05). All experiments were performed in triplicate and presented as the mean±SD statistical significance was performed using one-way ANOVA.

**Supplementary Table 1. Clinic pathological features of 50 CRC patients**

| Characteristics | | Total (%) |
| --- | --- | --- |
| Age (years) | < 65 | 34 (68) |
|  | >= 65 | 16 (32) |
| Gender | Female | 19 (38) |
|  | Male | 31 (62) |
| Tumor location | Rectum | 25 (50) |
|  | Colon | 25 (50) |
| Clinical stage | Ⅰ | 5 (10) |
|  | Ⅱ | 21 (42) |
|  | Ⅲ | 21 (42) |
|  | Ⅳ | 3 (6) |
| Lymph node metastasis (%) |  | 9 (18) |
| Distant metastasis (%) |  | 3 (6) |

**Supplementary Table 2: Primer sequences for Q-PCR**

| Gene | Primers (5`-3`) |
| --- | --- |
| CDCA5 | F: AGAAAGTCAGGCGTTCCTACAG  R: GGGAGATTCCAGGGAGAGTCAT |
| GAPDH | F: ATGGGGAAGGTGAAGGTCG  R: GGGGTCATTGATGGCAACAATA |

F, Forward；R, Reverse

**Supplementary Table 3. Correlation between CDCA5 expression and clinicopathological characteristics**

|  | Total (N=92) | CDCA5 protein expression | | 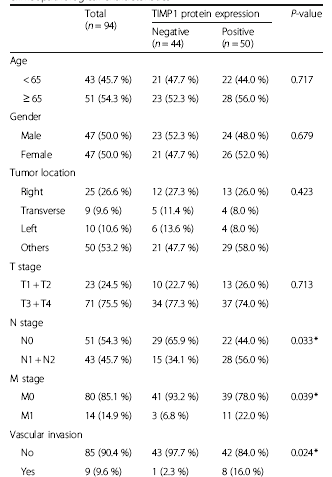P-value |
| --- | --- | --- | --- | --- |
|  |  | Low expression (n=9) | High expression (n=83) |  |
| Age |  |  |  |  |
| ≤ 65 | 38 (41.3%) | 5 (55.6%) | 33 (39.8%) | **0.032*** |
| ＞ 65 | 54 (58.7%) | 4 (44.4%) | 50 (60.2%) |  |
| Gender |  |  |  |  |
| Male | 51 (55.4%) | 6 (66.7%) | 45 (54.2%) | 0.321 |
| Female | 41 (44.6) | 3 (33.3%) | 38 (45.8%) |  |
| Pathology stage |  |  |  |  |
| Ⅰ | 2 (2.2%) | 1 (11.1%) | 1 (1.2%) | 0.255 |
| Ⅱ | 45 (48.9%) | 4 (44.4%) | 41 (49.4%) |  |
| Ⅲ | 45 (48.9%) | 4 (44.4%) | 41 (49.4%) |  |
| Tumor size |  |  |  |  |
| ≤ 5 cm | 46 (50%) | 4 (44.4%) | 42 (50.6%) | 0.420 |
| >5 cm | 46 (50%) | 5 (55.6%) | 41 (49.4%) |  |
| T stage |  |  |  |  |
| T1 | 5 (5.4%) | 0 (0%) | 5 (6.0%) | 0.124 |
| T2 | 6 (6.5%) | 2 (22.2%) | 4 (4.8%) |  |
| T3 | 69 (75%) | 6 (66.7%) | 63 (75.9%) |  |
| T4 | 12 (13.0%) | 1 (11.1%) | 11 (13.3%) |  |
| N stage |  |  |  |  |
| N0 | 58 (63.0%) | 9 (100%) | 49 (59.0%) | **0.000*** |
| N1 | 26 (28.3%) | 0 (0%) | 26 (31.3%) |  |
| N2 | 8 (8.7%) | 0 (0%) | 8 (9.6%) |  |
| M stage |  |  |  |  |
| M0 | 88 (95.7%) | 9 (100%) | 79 (95.2%) | 0.389 |
| M1 | 4 (4.3%) | 0 (0%) | 4 (4.8%) |  |
