# Supplementary figures and images for: Cell division cycle associated 5 promotes colorectal cancer progression by activating the ERK signaling pathway
Source: Oncogenesis. 2019 Feb 26;8(3):19. doi: 10.1038/s41389-019-0123-5 (PMC6391450; doi:10.1038/s41389-019-0123-5)

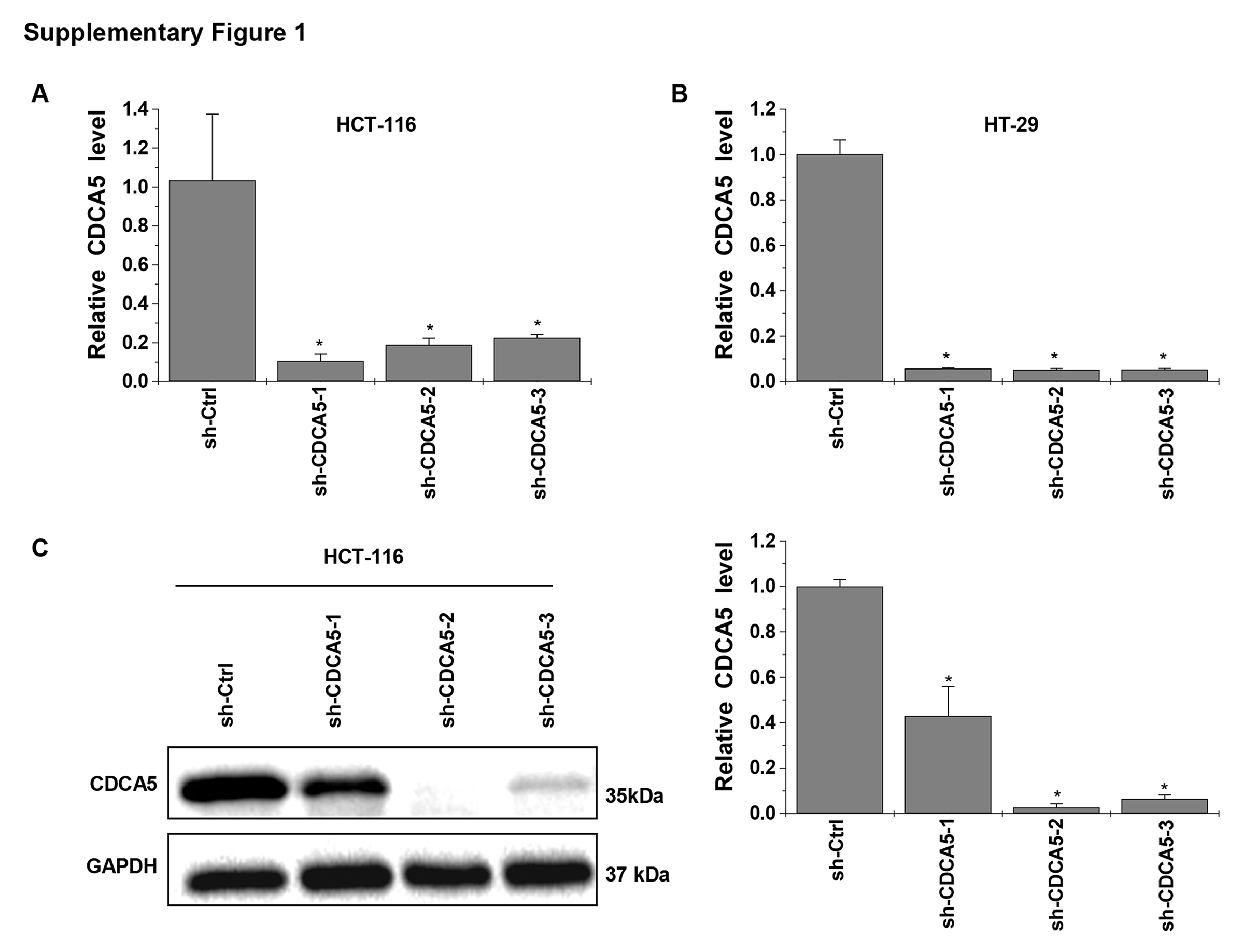

Supplement: Supplementary file 2 — Supplementary Figure 1. [file 41389_2019_123_MOESM2_ESM.jpg]
